# NK Cell-Mediated Antibody-Dependent Cellular Cytotoxicity in Cancer Immunotherapy

**DOI:** 10.3389/fimmu.2015.00368

**Published:** 2015-07-27

**Authors:** Wei Wang, Amy K. Erbe, Jacquelyn A. Hank, Zachary S. Morris, Paul M. Sondel

**Affiliations:** ^1^Department of Human Oncology, University of Wisconsin-Madison, Madison, WI, USA; ^2^Department of Pediatrics, University of Wisconsin-Madison, Madison, WI, USA

**Keywords:** natural killer cell, therapeutic monoclonal antibody, antibody-dependent cellular cytotoxicity, cancer, immunotherapy

## Abstract

Natural killer (NK) cells play a major role in cancer immunotherapies that involve tumor-antigen targeting by monoclonal antibodies (mAbs). NK cells express a variety of activating and inhibitory receptors that serve to regulate the function and activity of the cells. In the context of targeting cells, NK cells can be “specifically activated” through certain Fc receptors that are expressed on their cell surface. NK cells can express FcγRIIIA and/or FcγRIIC, which can bind to the Fc portion of immunoglobulins, transmitting activating signals within NK cells. Once activated through Fc receptors by antibodies bound to target cells, NK cells are able to lyse target cells without priming, and secrete cytokines like interferon gamma to recruit adaptive immune cells. This antibody-dependent cell-mediated cytotoxicity (ADCC) of tumor cells is utilized in the treatment of various cancers overexpressing unique antigens, such as neuroblastoma, breast cancer, B cell lymphoma, and others. NK cells also express a family of receptors called killer immunoglobulin-like receptors (KIRs), which regulate the function and response of NK cells toward target cells through their interaction with their cognate ligands that are expressed on tumor cells. Genetic polymorphisms in KIR and KIR-ligands, as well as FcγRs may influence NK cell responsiveness in conjunction with mAb immunotherapies. This review focuses on current therapeutic mAbs, different strategies to augment the anti-tumor efficacy of ADCC, and genotypic factors that may influence patient responses to antibody-dependent immunotherapies.

## Introduction

Natural killer (NK) cells have been described throughout the literature for their ability to kill virally infected and malignant cells without priming. Unlike B and T cells, NK cells do not require somatic gene rearrangements to produce highly specific receptors that recognize target cells (1, 2). Instead, mature NK cells reserve large amounts of cytotoxic granules containing perforin and granzymes, as well as the mRNA of IFNγ that is ready for translation if stimulated. As soon as the balance between inhibitory and activating signals within NK cells are skewed toward activation, NK cells are capable of forming synapses with target cells, allowing the release of the perforin and granzyme to lyse the target cells, as well as for IFNγ production (3). In addition, NK cells can initiate the transduction of death signals within target cells through death receptor/ligand ligation (4). Their capabilities of tumor cytotoxicity and inflammatory cytokine production enable NK cells to play an important role in different settings of cancer immunotherapy.

### NK cell recognition and “missing-self” hypothesis

Mature NK cells express a series of transmembrane receptors. The activating receptors allow them to recognize stress-induced ligands, while their inhibitory receptors prevent them from attacking normal cells. Activating receptors are often associated with adaptor proteins that have activation motifs in their cytoplasmic domains. Upon ligand binding, followed by phosphorylation, these activating receptors can activate down-stream kinases, leading to NK cell degranulation and cytokine secretion (5). Inhibitory receptors, on the other hand, have one or more inhibitory motifs in their cytoplasmic tails. Once phosphorylated, they recruit phosphatases and deactivate signaling kinases, resulting in NK cell inhibition (6). NK cell activity is tightly regulated through the balance between inhibitory and activating signals transduced by these receptors.

One family of receptors on human NK cells is that of the killer immunoglobulin-like receptors (KIR), which recognize HLA as their ligands (7). Some inhibitory and activating KIRs share the same ligand (8); however, most inhibitory KIRs have stronger binding affinity to their shared ligand (9, 10). One way to shift the activating-inhibitory balance toward NK cell activation is by decreasing the inhibitory KIR signaling. When cells are under stress or virally infected, the HLA expression on their surface is often downregulated in order to escape from T cell recognition. When an NK cell encounters these “target” cells, an immune mediated synapse can occur. If the target cell is missing the expression of the HLA ligand for the inhibitory receptors on that NK cell, and expresses ligands for the activating receptors, the interaction could lead to NK cell activation due to lack of inhibitory signals (“missing-self” hypothesis) (11, 12).

However, within an individual, not all NK cells have the same receptor expression profile, and each individual has a different KIR expression profile. As a result, not all NK cells express inhibitory KIR receptors (13, 14). The NK cells without any inhibitory KIR receptors have been shown to be hyporesponsive to HLA-null targets compared to NK cells expressing inhibitory KIR receptors that can recognize self-HLA molecules (15). NK cells that express no inhibitory KIR receptors, or KIR receptors that only recognize allogeneic HLA (i.e., which do not recognize or bind self-HLA) do not go through a “licensing” process during NK cell differentiation. Licensing plays a role in enabling the licensed NK cells to be more capable of killing targets that do not express inhibitory HLA ligands (such as HLA-null targets). Unlicensed NK cells are less potent in their activation by, or killing of, HLA-null targets, than are licensed NK cells (16). Interestingly, in mouse models, such unlicensed (hyporesponsive) NK cells, which express Ly49 receptors (the inhibitory receptors found on mouse NK cells that are the functional counterparts of human inhibitory KIRs) but have not seen “self-ligand,” can be made to be functional by transferring these NK cells to an environment where cognate ligand is expressed (17, 18).

Besides the expression of HLA ligands for the inhibitory receptors on NK cells, there are other mechanisms by which self-normal cells are protected from being attacked by NK cells. Studies in both mice (19–23) and humans (24, 25) have shown that after continuous exposure to the ligand for NK-activating KIR receptors, NK cells expressing these activating receptors can become hyporesponsive to target cells that express the ligand. This is evidence that the NK cells can be desensitized through their continual receptor contact with activating ligands.

### ADCC mechanism

In the setting of tumor-targeting monoclonal antibody (mAb) therapies, the anti-tumor efficacy of many mAb are shown to be NK cell-dependent (26). Human NK cells can express both FcγRIIC/CD32c (27) and FcγRIIIA/CD16a (28), which bind to the Fc portion of human immunoglobulins. FcγRIIIA often associates with FcϵRI-γ chains or CD3-ζ chains within the cell membrane, or with a heterodimer of these two chains (5). Both FcϵRI-γ and CD3-ζ chains have immune tyrosine-based activating motifs (ITAM) in their cytoplasmic tails. Unlike most activating receptors on NK cells, FcγRIIC has an ITAM in its own cytoplasmic tail. Upon FcγR binding, these ITAMs are phosphorylated, and through signal transduction mechanisms (binding to tyrosine kinases ZAP-70 and Syk and activation of PI3K, NF-κb and ERK pathways) NK cell degranulation, cytokine secretion, and finally tumor cell lysis occur (29).

Antibody-dependent NK-mediated tumor killing occurs through several different pathways, including: (1) exocytosis of cytotoxic granules; (2) TNF family death receptors signaling; (3) pro-inflammatory cytokine release, such as IFNγ. Both the uptake of perforin and granzymes by target cells and TNF family death receptor signaling cause target cell apoptosis (29), while IFNγ released by NK cells activate nearby immune cells to promote antigen presentation and adaptive immune responses (30). IFNγ production and cytotoxicity have been considered two distinctive functions of different NK subsets (31, 32), but growing evidence shows that the main cytotoxic NK subset, CD56^dim^CD16+ NK cells, that are responsible for mAb-mediated tumor killing, are also able to produce IFNγ following activation (33, 34). In addition to inhibiting cell proliferation, angiogenesis, and increasing MHC surface expression (35), IFNγ was also shown to contribute to upregulation of TRAIL expression on NK cells (36), which suggests that one mechanism may interact with another to synergistically enhance tumor killing. A recent study indicates that NK-insensitive targets can become NK-sensitive via treatment with IFNγ, which induces lysis through ICAM-1 upregulation and increasing conjugate formation with NK cells (37). These mechanisms might work together to eliminate tumor targets through engagement of both innate and adaptive immunity; whether one is predominant over the others in tumor killing is still unknown (29–37).

### NK cell FcγRs in ADCC

Genotypic variations (polymorphisms) exist in humans in both FcγRIIIA and FcγRIIC that influence FcR function. Thus, FcγRIIIA and FcγRIIC genotype can influence the interaction of these receptors with immunoglobulin, resulting in differential effectiveness of mAb therapy depending on an individual’s genotype. In addition, immunoglobulin isotypes (IgG1, IgG2, IgG3, and IgG4), as well as fucosylation and glucosylation patterns, have varying influence on the affinities of these IgG molecules for both FcγRIIIA and FcγRIIC (38–41). Such factors (patient FcγR genotype and antibody Fc backbone) create the opportunity for considering therapeutic treatment options that may optimize the degree to which a patient will respond when administering mAb therapy. Selection of an optimized regimen may lead to a more effective trigger of the proper immune response.

A number of studies have shown that anti-tumor activity of certain tumor-specific mAbs is associated with higher affinity FcRs, based on the FcR genotype. These results suggest that these mAbs are acting through antibody-dependent cell-mediated cytotoxicity (ADCC) by the cells that express those FcRs. In particular, FcγRIIIA expressed on NK cells has a single nucleotide polymorphism (SNP) that results in FcγRIIIA polymorphic variants [FcγRIIIA with phenylalanine (F) at amino acid position 158, or FcγRIIIA with valine (V) at amino acid 158], which vary in their strength of binding to immunoglobulins. Several studies have shown that those individuals that are homozygous for V (FcγRIIIA-158-V/V) have improved clinical outcome (after treatment with ADCC-inducing tumor-reactive mAb) over those that are either heterozygous (FcγRIIIA-158-V/F) or homozygous (FcγRIIIA-158-F/F) for the lower affinity FcγRIIIA isoform (42–46). While not all such studies confirm these findings, several clinical analyses are consistent with this result. These are presented in the section below regarding a few of the mAbs that appear to be acting, at least in part, via ADCC.

## Therapeutic Monoclonal Antibodies for Cancer Treatment

Tumor-specific mAbs that recognize tumor-selective antigens on the surface of tumor cells are being used as cancer therapy. These therapeutic mAbs target and attack tumor cells through various mechanisms, including directing toxic molecules to target cells, inhibiting target cell proliferation, blocking inhibitory signals for immune cells, and directing immune cells to kill targets through ADCC (47). Some newer antibodies, such as bi-specific antibodies (bsAbs), work through promoting conjugate formation between target cells and NK or T cells (48). A more comprehensive list of anti-cancer mAbs has been summarized in other reviews (47, 49, 50). This review focuses on NK cell-mediated anti-tumor activity, via ADCC, and thus includes discussion of representative tumor-specific mAbs that have been shown in pre-clinical or clinical models to function, at least in part, via ADCC (Table 1).

**Table 1:** **Representative tumor-antigen targeting monoclonal antibodies and immunocytokines functioning through ADCC**.

	Target	Status	Reference or clinical trial#
**MAb**			
Rituximab	CD20	FDA approved for non-Hodgkin’s lymphoma	Cartron et al. (42)
		Phase I relapsed indolent B-cell non-Hodgkin	NCT02384954
		Lymphoma, combined with ALT-803^a^	
		Combination with matrix metalloproteases inhibitor in pre-clinical models	Romee et al. (51)
Obinutuzumab	CD20	FDA approved for chronic lymphocytic leukemia	Goede et al. (52)
Hul4.18K322A	GD2	Phase I neuroblastoma, melanoma,	NCT01576692
		Osteosarcoma, ewing sarcoma	NCT00743496
		Phase II neuroblastoma	NCT01857934
Hu3F8	GD2	Phase I GD2+ tumors	NCT01419834
		Phase I high-risk neuroblastoma and GD2+ solid	NCT01662804
		Tumors, combined with IL2	
		Phase I refractory high-risk neuroblastoma, combined with GM-CSF	NCT01757626
Dinituximab	GD2	FDA approved for high-risk neuroblastoma, combined with IL2 and GM-CSF	Yu et al. (53)
Trastuzumab	HER2	FDA approved for HER2+ breast cancer and HER2+ metastatic gastric adenocarcinoma	Junttila et al. (54)
Cetuximab	EGFR	FDA approved for metastatic colorectal cancer and head and neck cancer	Messersmith and Ahnen (55)
**IMMUNOCYTOKINE**			
Rituximab-RLI^b^	CD20	Tested for human B lymphoma in SCID mouse	Vincent et al. (56)
c.60C3-RLI^c^	GD2	Tested in mouse GD2+ cell lines EL4 (subcutaneous) and NXS2 (metastatic)-grafted mouse models	Vincent et al. (57)
Hul4.18-IL2	GD2	Completed phase II refractory neuroblastoma	Delgado et al. (58), Shusterman et al. (59)
KM2812	PSMA	Tested in human prostate cancer cell LNCaP-xenografted mouse model	Sugimoto et al. (60)
**BI-SPECIFIC ANTIBODY AND SINGLE CHAIN VARIABLE FRAGMENT**			
AFM13	CD30/CD16	Phase II relapsed Hodgkin lymphoma	NCT02321592
(CD20)_2_xCD16	CD20/CD16	Tested in humanized mouse grafted with autologous human B cells	Glorius et al. (61)

*^a^ALT-803 is a fusion protein consisting of mutated IL15 and IL15Rα/Fc complex*.

*^b^RLI (IL15Rα-linker-IL15) is a fusion protein linking the NH_2_-terminal domains of IL15Rα to IL15 through a 20-amino acid linker*.

*^c^c.60C3 is a chimeric anti-GD2 mAb*.

*This table is a selected (not complete) list of therapeutic mAbs that are capable of inducing antibody-dependent cellular cytotoxicity*.

### Anti-GD2 mAb for melanoma and neuroblastoma treatment

GD2 is a disialoganglioside expressed on human melanoma and neuroblastoma cells with restricted expression on normal tissues, which makes it a suitable target for mAb immunotherapy (Table 1). The first anti-GD2 antibody, 3F8, is a murine IgG3 mAb that was produced in 1985 from a mouse hybridoma (62). 3F8 is able to elicit complement activation and ADCC against human neuroblastoma cells. However, most patients in early clinical trials that received 3F8 developed human anti-mouse antibody (HAMA) response (63). HAMA may compete for the binding site of the therapeutic antibodies resulting in decreased binding to GD2, therefore dampening the anti-tumor efficacy and leading to acceleration of clearance of the therapeutic antibody from circulation (64).

Another murine anti-GD2 mAb, 14.18, which is also an IgG3, was generated separately by Mujoo et al. in 1987 (65). This antibody also displayed the capability to induce efficient *in vitro* ADCC and *in vivo* anti-tumor effects. An isotype variant of this murine anti-human GD2 antibody, 14.G2a (66), was tested clinically and showed some anti-tumor activity (67, 68), but HAMA response was still present in a significant portion of patients. While effective in targeting tumor and reducing tumor size in occasional patients, it became evident that it was necessary to improve the backbone of these initial mAb to increase efficacy and decrease the immunogenicity of this immunotherapeutic option.

In order to reduce the HAMA response and lengthen the antibody half-life in patients, efforts were made to create chimeric anti-GD2 antibodies, containing human constant regions with murine variable regions. Since a chimeric antibody has a majority of human epitopes, these epitopes should not be recognized by the immune system as foreign, and thus be less immunogenic than the fully murine antibodies. Dinituximab (formerly known as ch14.18) is a chimeric mAb comprising a fusion protein of the human constant portion of IgG1 and the GD2-reactive variable portion of the murine 14.18 mAb (69). Dinituximab has been shown to induce stronger ADCC than 14.G2a *in vitro* against GD2-positive neuroblastoma cells (70), and have anti-tumor activity against GD2-positive melanoma cells *in vivo* (71). In the initial published phase I clinical study of dinituximab treatment for pediatric neuroblastoma (72), no human anti-chimeric antibody (HACA) response was detected. Four out of nine children had anti-tumor response and one had a minor response. Thus, by modifying the backbone of the antibody, improved clinical outcome was observed.

To further improve antibodies, a fully human antibody was “grafted” with murine complementarity determining regions (CDRs), which confer antigen specificity. These humanized antibodies are considered less immunogenic than chimeric antibodies (73). However, even with humanized antibodies specific for GD2, pain and capillary leak were seen as significant toxicities. These toxicities limit the dose that can be administered, which restrains the possible anti-tumor effect that one would expect if a higher dose could be given. The toxicities are mainly attributed to complement activation (74), which is elicited by the CH_2_ domain on antibodies (75). Therefore, by reducing complement activation via a point mutation at amino acid position 322 in the CH2 domain of humanized antibody, complement activation is greatly reduced. Such reduction in complement activation, and thus reduced toxicities (76), allowed for higher treatment-dose to be administered to patients, while at the same time maintaining the anti-tumor ADCC effect (77). Both humanized 14.18K322A and humanized 3F8 are under clinical investigation (Table 1) (73, 78).

### Herceptin/trastuzumab

Trastuzumab is a humanized anti-HER2 mAb used to treat HER2-positive breast carcinoma (Table 1), as well as many other types of cancers that overexpress HER2, a member of the human epidermal growth factor receptor (EGFR) family. HER2 is a transmembrane tyrosine kinase with no known ligand. Dimerization of HER2 with certain EGFR family members leads to activation of signaling pathways that promote cell proliferation and survival (79). HER2 is overexpressed on a variety of tumors with limited expression on normal tissues, thus it is an ideal target for treatment of HER2-positive cancers.

Trastuzumab was first approved by the FDA in 1998 to treat HER2-positive metastatic breast cancer. Besides preventing HER2 from dimerization, trastuzumab was also shown to mediate ADCC against HER2-positive tumor cells *in vitro*, and the major effector cells were NK cells expressing FcγRIIIA (80, 81). A mutant trastuzumab that lost the ability to bind to FcγR lost anti-tumor activity *in vivo* in a xenograft breast tumor model, suggesting that ADCC is involved in the anti-tumor effect of anti-HER2 mAb therapy *in vivo* (54). In addition, Clynes et al. showed that the anti-tumor response to trastuzumab in a breast carcinoma xenograft mouse model was decreased in mice lacking the activating receptor FcγRIIIA, but enhanced in mice lacking the inhibitory receptor FcγRIIB (82). These experimental data demonstrate that Fc receptor recognition is responsible for at least part of anti-tumor efficacy of trastuzumab.

### Cetuximab

Cetuximab is an FDA-approved chimeric mAb for treatment of EGFR-expressing metastatic colorectal cancer (mCRC) (55), metastatic non-small cell lung cancer, and head and neck cancer. It reacts against the human EGFR, and can interfere with tumor growth via receptor blockade from growth factor activation. *In vitro* studies indicate that some of the anti-tumor activity of cetuximab is mediated via ADCC (83, 84), and cetuximab-mediated *in vitro* ADCC is correlated with NK cell FcγR polymorphisms of the effector donors (46, 85). In addition, in mCRC patients treated with cetuximab and irinotecan, those who have higher affinity FcγR polymorphisms had longer progression-free survival (45, 86). In other studies of mCRC patients, the opposite association has been found, namely low affinity FcγR polymorphism was associated with better clinical outcomes (87–89). However, the patients in these studies either had different percentage of KRAS mutation or received other antibody concurrently with cetuximab, which indicates patient mutation profile as well as treatment regimen could also influence the impact of FcγR on cetuximab response (90). Nevertheless, both *in vitro* and some clinical data suggest that cetuximab-mediated ADCC through NK cells may contribute to its anti-tumor activity.

### Rituximab and obinutuzumab

Rituximab is a chimeric IgG1 mAb targeting CD20, a B cell differentiation antigen (Table 1). There are various mechanisms that may account for the anti-tumor effect of rituximab: complement-dependent cytotoxicity (CDC), direct target cell apoptosis, antibody-dependent phagocytosis and ADCC (91). In xenograft mouse models of B-cell lymphoma, the anti-tumor effect of rituximab was greatly reduced in FcRγ^−/−^ nude mice (82), or in mice treated with FcγR block (92). Interestingly, clinical evidence gathered by Cartron et al. suggested that FcγRIIIA on NK cells plays an important role in anti-tumor effect of rituximab. Cartron et al. evaluated FcγRIIIA polymorphisms in follicular lymphoma patients treated with rituximab, and this was the first time that better response to rituximab was associated with higher affinity FcγRIIIA genotype (42). However, there have been mixed results as to the influence of FcR genotype on clinical response to rituximab. Some groups have found in B cell lymphoma patients with no association between FcγR polymorphism and outcome (93–95). There are various factors that could contribute to the differences in these findings, including patient population, genotyping methodology, rituximab treatment strategy, and whether or not patients received concurrent chemotherapy (96–99).

Modifications have been made to improve the binding affinity of therapeutic anti-CD20 mAb to activating Fc receptors, in hopes of maximizing the ADCC function of this mAb therapy. One such effort consists of using glycoengineered antibodies, which are produced in CHO cells that overexpress β-1,4-N-acetyl-glucosaminyltransferase III and Golgi α-mannosidase II, and will increase the binding affinity to both the higher affinity and to the lower affinity isoforms of FcγRIIIA (39, 41, 100). The first Fc-glycoengineered anti-CD20 humanized mAb, obinutuzumab, was shown to induce stronger ADCC and direct target cell death *in vitro*, and it also elicited better anti-tumor activity in a lymphoma xenograft mouse model compared to rituximab (101, 102). In a phase III clinical trial comparing obinutuzumab vs. rituximab, combined with chemotherapy, for treating chronic lymphocytic leukemia (CLL) patients (52), obinutuzumab plus chlorambucil significantly prolonged progression-free survival compared to rituximab plus chlorambucil. Obinutuzumab alone, or in combination with chemotherapy, is currently under clinical investigations for other B-cell malignancies as well (103–106).

## How to Augment Anti-Tumor Effects of ADCC

Since ADCC is an important contributor to the anti-tumor activity of many mAb therapies, enhanced immune activation of the effector cells may be an ideal adjuvant therapy to augment ADCC activity of mAb. In addition to the ADCC capabilities of NK cells, they can also stimulate the activity of other immune processes through their release of cytokines (such as IFNγ), and thus can provide a link to initiate additional immune responses to attack target tumors.

### mAb + radiation therapy

Approximately 60% of oncology patients receive radiation therapy as part of their cancer treatment. Radiation elicits an anti-tumor effect through the induction of DNA damage but may also increase tumor susceptibility to immune response (107). Consequently, the effect of radiation may be modulated by immune response (108, 109) and radiation may augment the efficacy of immunotherapies (110). The mechanisms by which radiation may interact with the immune system include radiation-induced production of inflammatory cytokines, release of tumor-specific antigens, phenotypic changes in tumor cell expression of immune susceptibility markers, and effects on vascular architecture that enhance immune surveillance (107, 110). Included among the phenotypic changes induced by radiation are the upregulation of MHC class I, NKG2D ligand, and the Fas death receptor; all of which may potentiate the ADCC response (111–114). In addition, radiation may impact the expression of antigens targeted by tumor-specific antibodies and this has been show to enhanced ADCC response *in vitro* (115, 116). The potential interaction of radiation and ADCC has not yet been clarified *in vivo*. Interestingly, however, a number of tumor-specific antibodies that are known to elicit ADCC (including cetuximab, trastuzumab, and dinituximab) are commonly administered to patients that also receive radiation therapy. The role of ADCC and NK cells in the clinical response to such combined modality treatment has not been defined. Further pre-clinical investigation is needed to evaluate whether the effects of radiation on tumor immune susceptibility may be leveraged to enhance ADCC response in the clinical setting.

### mAb + matrix metalloproteases inhibitor

Upon activation, NK cells have been shown to shed FcγRIIIA (also known as CD16) from their cell membrane, a process that is mediated by matrix metalloproteases (MMPs) activation (117, 118). *In vitro* treatment of NK cells with the MMP inhibitor, GM6001, rescues FcγRIIIA loss stimulated by K562 tumor cells, but does not interfere with NK cell degranulation (118), which indicates that FcγRIIIA shedding and degranulation of NK cells are dependent upon separate pathways. It is plausible, therefore, that maintaining FcγRIIIA expression on NK cell surface via an MMP inhibitor could enhance the ADCC function of NK cells without interrupting NK cell degranulation (119). Recently, one specific MMP, ADAM17, was identified by Romee et al. as the key MMP responsible for FcγRIIIA expression loss after NK cell activation (51). According to their report, an ADAM17-specific inhibitor not only rescues FcγRIIIA shedding stimulated by tumor targets but also improves NK cell degranulation as well as IFNγ production in the presence of tumor-specific mAb (51). These *in vitro* studies suggest that limiting the loss of FcγRIIIA on the cell surface is important for enhanced NK cell-mediated ADCC, and opens new possibilities for combination of both MMP and mAb therapies to further promote ADCC.

### Anti-GD2 mAb + IL2 + GM-CSF

While anti-GD2 mAb alone produced some anti-tumor activity in neuroblastoma patients, combining the mAb with GM-CSF and interleukin 2 (IL2) to further activate immune effector cells enabled potent clinical anti-tumor efficacy of anti-GD2 mAb. Our lab previously showed that peripheral blood mononuclear cells (PBMCs) obtained from cancer patients pre-IL2 treatment had low levels of ADCC against a neuroblastoma cell line, even in the presence of both anti-GD2 mAb and IL2. However, PBMCs obtained from the same patient following 4 weeks of IL2 infusions mediated much higher ADCC of neuroblastoma cells, and the addition of IL2 *in vitro* dramatically boosted the anti-GD2 mAb-mediated ADCC (120, 121). Moreover, depletion of FcγRIIIA-positive cells eliminated ADCC completely (120). These data suggest that *in vivo* infusion of IL2 could overcome the immune suppression seen in some cancer patients; when using NK cells obtained from patients following therapy with IL2, the *in vitro* combination of IL2 and anti-GD2 mAb greatly boosted ADCC of neuroblastoma cells.

Besides IL2, GM-CSF also acts as an immune stimulator, especially following immune suppressive chemotherapy, to rescue bone marrow myeloid function (122–125). The combination of GM-CSF and the murine anti-GD2 mAb, 3F8, resulted in complete responses of GD2-positive cancer patients, particularly in patients with minimal residual disease (MRD) (126). In a phase III clinical trial for high-risk neuroblastoma patients following induction therapy and autologous transplant, patients were randomized into standard therapy (isotretinoin) or into a group that received the combined immunotherapy regimen of dinituximab + IL2 + GM-CSF in addition to isotretinoin. Patients in the immunotherapy treatment group had significantly improved event-free survival and overall survival as compared to the patients that received the standard of care alone (53). Other mAb therapies, such as rituximab and trastuzumab (81), are also being combined with IL2 in clinical trials to evaluate the anti-tumor efficacy (127–129).

### mAb + IL15

IL15 is another cytokine that can activate immune cells, such as NK and T cells. IL15 receptors (IL15R) share the same β chain and common γ chain as IL2 receptors, but IL15R have a unique α chain that is specific for IL15, which is used for IL15 presentation to β/γ receptors on immune cells. Soluble IL15 has been shown to activate NK cells, enhance NK-mediated cytotoxicity, and cytokine production *in vitro* (130), and administration of recombinant human IL15 (rhIL15) to cancer patients resulted in *in vivo* NK cell proliferation and activation (131). While IL2 has the potential for T regulatory cell (Treg) maintenance, which could dampen activating immune response, IL15 does not stimulate Tregs (132). Moreover, IL15 does not trigger T cell death after activation, or lead to vascular leak syndrome (VLS), both of which are significant toxicities seen in pre-clinical models using IL2 (133–135). Therefore, in addition to IL2, IL15 is a potential candidate that can be used clinically combined with mAb, to improve NK cell-mediated ADCC without severe toxicity.

Indeed, the Caligiuri group reported enhanced NK-mediated ADCC by IL15 in 1994 (130). More recently, Moga et al. showed that *in vitro* activation of PBMCs by IL15 significantly improved rituximab-mediated ADCC against B lymphoma cells, and the major effector cells were NK cells (136). They also showed this enhancement of rituximab-mediated ADCC using PMBCs from CLL patients against a CLL cell line (137). In addition, they showed that IL15 treatment resulted in similar ADCC levels between individuals with the lower affinity FcγRIIIA and individuals that have the higher affinity FcγRIIIA (137). These findings suggested that the difference between the capabilities of the high and low affinity FcγRIIIA to mediate ADCC might be overcome by IL15 treatment. Such findings suggest that IL15 administration may compensate for mAb-affinity differences that are dependent upon FcγRIIIA polymorphisms.

Different from IL2, the activation of NK and T cells by IL15 is through trans-presentation by the IL15Rα chain, which is expressed independent of the β/γ chains. With efforts to increase the stimulation activity of soluble IL15, several different IL15 agonists have been generated and tested in pre-clinical models in order to boost NK-mediated ADCC efficacy. A fusion protein linking IL15Rα chain sushi domain and human IL15 (RLI) has been generated and shown superior stimulation potential *in vitro* and better anti-tumor effects *in vivo* than soluble IL15 (138, 139). In addition, an IL15 mutant has been identified as a better surrogate for soluble IL15 due to its enhanced activity to stimulate proliferation of cells expressing IL15R (140). Later on, the same group generated a fusion protein consisting of the IL15 mutant and an IL15Rα/Fc complex (141). This IL15 superagonist, ALT-803, could improve rituximab-mediated ADCC against B cell lymphoma both *in vitro* and *in vivo* [Maximillian (142)]. Thus, IL15 combined with tumor-specific mAb appears to merit clinical testing as a potential immunotherapy, which is currently underway (NCT02384954) (Table 1).

### Immunocytokines

#### Anti-GD2 Immunocytokines

Immunocytokines (IC) are fusion proteins made by linking immune-activating cytokine to a tumor-specific mAb. The initially described IC (ch14.18-IL2) linked IL2 to the C-terminus of the ch14.18 chimeric anti-GD2 mAb (143). The IL2 component on these IC has the same ability as soluble IL2 in terms of stimulating cell proliferation via IL2 receptors (IL2R) (144), and have been shown to mediate the conjugation between IL2R-positive cells and tumor target cells (145). IC also preserve FcR binding ability, and thus are capable of mediating *in vitro* ADCC (144). By targeting IL2 to the tumor microenvironment, IC might be superior at activating tumor infiltrating immune cells resulting in improved ADCC while causing less toxicity than soluble IL2. Lode et al. showed that ch14.18-IL2 had improved anti-tumor efficacy than the combination of ch14.18 and soluble IL2 in a spontaneous neuroblastoma metastases mouse model (146). They also showed that the mechanism behind this anti-tumor effect strongly depended on NK cells, since NK cell depletion completely abrogated the anti-tumor effect in this model (147).

A separate humanized IC was created by linking human IL2 to the humanized 14.18 mAb (hu14.18-IL2; Table 1). This IC was assessed for anti-tumor activity in a phase II clinical trial of refractory neuroblastoma patients divided into two strata of patients depending on disease burden. Stratum 1 included patients with disease measurable by computed tomography and/or magnetic resonance imaging using standard radiographic criteria, while stratum 2 included patients with disease evaluable only by ^123^I-MIBG scintigraphy and/or BM histology. Of 36 patients evaluable for response, no responses were seen in the 13 patients in stratum 1 while 5 complete responses were noted in the 23 patients in stratum II (59). In another phase II clinical trial of hu14.18-IL2 in 14 patients with measurable metastatic melanoma, 1 patient had a partial response to the immunotherapy (148). Both of these trials suggest that hu14.18-IL2 works better in clinical cases where MRD is present than in individuals that have bulky disease. Furthermore, in the phase II neuroblastoma study of hu14.18-IL2 noted above where 21.7% of stratum II patients with MRD responded, all of the responders had a favorable KIR/KIR-ligand genotype (i.e., KIR-ligand missing) (58). This study indicates that NK cells play an important role in response to the hu14.18-IL2 treatment in neuroblastoma patients.

#### Anti-PSMA Immunocytokine

Prostate-specific membrane antigen (PSMA) is a surface antigen highly expressed by poorly differentiated prostate cancers, which makes it a suitable target for mAb therapies. Several anti-PSMA mAb and antibody-drug conjugates have been tested clinically, and a small fraction of patients showed anti-tumor response, which in part was due to ADCC (149). To improve ADCC function of anti-PSMA mAb, an anti-PSMA IC was generated by fusing IL2 to a mouse/human chimeric anti-PSMA mAb (60). This IC showed enhanced *in vitro* ADCC, and superior *in vivo* anti-tumor activity compared to a naked anti-PMSA mAb (60). Continued research is underway in both pre-clinical models and clinical testing to determine if superior anti-tumor efficacy is noted with this novel IC.

#### IL15-Linked Immunocytokine

One limitation of IL2-linked ICs is IL2-mediated toxicity that restricts the maximum-tolerated dose (MTD) that can be administered. In contrast to IL2, IL15 does not appear to elicit as severe adverse side effects. In addition, IL15 may have improved anti-tumor efficacy (133). Since IL15 is most active in a trans-presentation form, a fusion protein of human IL15Rα and human IL15, called RLI, was generated. This fusion protein showed superior activation function than soluble IL15 both *in vitro* and *in vivo* (138, 150). Vincent et al. linked RLI to the end of chimeric anti-GD2 antibody (c.60C3) and generated an RLI-linked anti-GD2 IC (57). This IC had similar IL15-induced proliferative activity as RLI, and similar ADCC-inducing ability as anti-GD2 antibody *in vitro*. Moreover, anti-GD2-RLI exhibited better anti-tumor activity than either RLI or anti-GD2, alone or in combination, in an NXS2 mouse neuroblastoma model (57). The same group also showed that RLI-linked rituximab significantly prolonged survival as compared to administration of RLI and rituximab at the same time in a residual lymphoma mouse model (56). These pre-clinical data suggest that IL15-linked IC merit clinical investigation to evaluate both efficacy and toxicity relative to IL2-linked IC.

### Novel bi-specific antibodies or single chain variable fragment targeting NK cells

With evolving genetic engineering technologies, non-conventional antibodies that have dual or tri specificity have been constructed. They either target two different antigens on tumor cells, or facilitate conjugate formation between immune cells and tumor targets (151). There are two classes of bsAbs: Fc-containing bsAbs that are of similar size as conventional mAbs and those bsAbs without an Fc domain, which are of much smaller size. They are further divided according to their structures, specificities, or how they are constructed (152). In efforts to bring NK cells and tumor cells in proximity, several bsAbs or single chain variable fragment (scFv) have been constructed to bind both tumor antigen and FcγRIIIA (CD16). AFM13 is a bi-specific tetravalent antibody construct that targets both CD30 and CD16 (153), which is currently in a phase II clinical trial for relapsed Hodgkin lymphoma patients (NCT02321592) (Table 1). It has been shown to exhibit superior cytotoxicity than other CD30-targeting antibodies *in vitro* and its ADCC activity is independent of the FcγRIIIA allotypes (154). A CD20/CD16 bsAb also showed improved clearance of B cell malignancy compared to rituximab both *in vitro* and *in vivo*. Again, the efficacy of this antibody construct is not influenced by FcγRIIIA polymorphism (61). Two different CD33/CD16 bsAbs have been shown to efficiently kill acute myeloid leukemia (AML) cells or stem cells from myelodysplastic syndrome, a precursor of AML (155–157). A few CD19/CD16 bsAb or derivatives have also been generated to target leukemia and lymphoma cells and showed promising ADCC activity *in vitro* (158–161).

For solid tumors, a bsAb-targeting HER2/CD16 has been tested in mouse model against HER2-positive tumor cells and showed enhanced anti-tumor efficacy than trastuzumab against HER2-low expressing tumor. Interestingly, the efficacy of this bsAb is also FcγRIIIA polymorphism independent (162). In addition, an EpCAM/CD16 bi-specific scFv antibody fragment showed enhanced *in vitro* killing of human carcinomas with a broad range of origins, as well as efficient killing of NK-resistant carcinoma targets (163). The fact that a lot of these bsAbs discussed above have proficient anti-tumor effect regardless of FcγRIIIA affinity on NK cells enlarges the patient population that will potentially benefit from such type of immunotherapy.

### Adoptive transfer of *Ex Vivo*-activated NK cells

A separate approach to augment NK cell function and ADCC is to infuse NK cells that have been activated and expanded in cell number *ex vivo*. During the *ex vivo* expansion of NK cells, these activated effectors are primed to kill tumors more effectively, and they can then be infused into cancer patients. Autologous NK cells may be relatively tolerant to self-tumors and, in certain settings, may have less anti-tumor potential than allogeneic NK cells (164). There are several approaches being pursued in order to expand and activate NK cells *ex vivo*. In the presence of IL2 and irradiated feeder cells, such as EBV-LCL cells or genetically modified K562 cells, NK cells are preferentially expanded to 200- to 400-fold within a 21-day period (165, 166). NK cells can be potently expanded and activated by culturing them with K562 cells that have been modified to be far more stimulatory by expressing membrane-bound IL15 and 41BBL. NK cells that have been *ex vivo* expanded using these modified K562 cells have better anti-tumor activity *in vitro* and *in vivo* in mouse models (166). In addition, *ex vivo*-expanded NK cells have also been shown to be able to mediate ADCC, and in combination with tumor antigen-specific antibodies, they exerted better anti-tumor efficacy (167, 168). Different protocols using expanded NK cells are currently under clinical testing, either alone or in combination with tumor-specific mAb (169–171). These ongoing clinical trials may answer the questions of how long these NK cells may survive *in vivo*, their homing capacity, and tumor-targeting specificity.

## Genotypic Factors That Affect Antibody/NK Cell Based Immunotherapy

Despite the success noted in some immunotherapies involving NK cells, some patients do not benefit from these immunotherapies. Therefore, it would be advantageous to be able to identify those patients who would likely respond, and those who would be less likely to benefit from immunotherapies that require NK cell response. There are many different genotypic factors that could potentially affect the efficacy of different immunotherapies in various cancer types. Here, we focus mainly on those genotypes that could affect NK cell function and ADCC efficacy.

### Killer-immunoglobulin receptors on NK cells and their ligands

Killer-immunoglobulin receptors are expressed on a subset of NK cells and on some T cell subsets. KIRs are a family of receptors with high polymorphisms inherited on chromosome 19 (172). There are 15 functional KIR genes and 2 KIR pseudogenes in the human genome. Of the functional KIR genes, some function as inhibitory receptors and others are activating receptors (173). For some KIR genes, the ligands are well defined (7). KIRs with defined ligands generally recognize distinct HLA class I molecules, but do so with less specificity than T cell receptors. Furthermore, and again in distinction to T cell receptors, KIRs appear to recognize class I HLA molecules without regard to peptides that may be presented in the cleft (pocket) of the HLA structure. Some inhibitory and activating KIRs with high homology in extra cellular domains share the same HLA ligand, with the inhibitory KIR/KIR-ligand interaction being a much stronger binding pair. The interaction between KIRs and KIR-ligands influence NK cell education and function (15, 174). Since KIR haplotype, as well as KIR expression, is highly diverse among the population, and since KIR-ligands are inherited independently from KIRs, an individual’s KIR/KIR-ligand genotype can affect NK cell function and ADCC in different settings. These differences in genotypes for KIR/KIR-L between different individuals have been associated with the individual’s outcome in response to certain forms of immunotherapy, particularly those immunotherapies that involve NK cells.

The KIR haplotype of NK cell donors (175–177) as well as the relationship between the donor’s inhibitory KIR and the recipient’s KIR-ligand genotypes can predict clinical outcomes in allogeneic hematopoietic transplantation for AML patients; the impact of KIR and KIR-L genotypes may also play a role in some, but not all, settings of hematopoietic transplantation for acute lymphoid leukemia (ALL) patients (178–180). Recipients that are missing the KIR-ligands for the KIR genes that are present on the donor NK cells are predicted to have longer progression-free survival post-transplantation than those who have the KIR-ligand genes present for the donor KIR genes (178–180). In an autologous hematopoietic transplantation setting for high-risk neuroblastoma patients, Venstrom et al. showed that patients who were missing any self-inhibitory KIR-ligand had improved progression-free survival and improved overall survival (181). Even though direct ADCC was not involved in these transplants for cancer patients, these data involving KIR/KIR-L relationships indicate that NK cells likely contribute to the anti-tumor response post-transplantation.

In some immunotherapies where ADCC is involved, patients missing their self-inhibitory KIR-ligand also had better clinical outcome. In neuroblastoma patients treated with anti-GD2 mAb or IC, and in lymphoma patients treated with rituximab, inhibitory KIR-ligand missing was associated with improved clinical outcome (58, 181–183). These findings suggest that self KIR/KIR-ligand genotypes not only affect NK cell function but also affect NK-mediated ADCC effects in the clinical setting.

The effects of inhibitory KIR/KIR-ligand interactions on NK cell function have been investigated for over a decade, but the role of activating KIR/KIR-ligand interactions in immunotherapies involving NK cells has only recently been reported. In 2011, Scquizzato et al. evaluated the impact of recipient KIR-ligand missing for both inhibitory and activating donor KIR genes, in a group of patients with various hematopoietic malignancies who received allogeneic hematopoietic stem cell transplantation (184). They found that the cohort of recipient patients missing the KIR-ligand for donor KIR genes had better disease-free survival, but only when patients who had KIR-ligands missing for their activating KIR were excluded from the analysis (184). This finding suggests that activating KIR/KIR-ligand interactions may have differential impact on patient outcome than inhibitory KIR/KIR-ligand interactions, thus the KIR-ligand missing model to predict patient outcome appears to be applicable only to inhibitory KIRs, with exclusion of activating KIRs.

In 2012, Venstrom et al. reported that in a large group of AML patients, those who received allografts from donors that had the activating KIR gene, KIR2DS1, and two copies of the ligand for KIR2DS1, HLA-C2, exhibited a higher relapse rate than patients who received grafts from KIR2DS1-positive donors and either one or no copies of HLA-C2 (24). Consistent with this clinical observation, Pittari et al. showed that KIR2DS1-positive NK cells from healthy donors that have two copies of HLA-C2 were hyporesponsive to tumor targets as compared to KIR2DS1-positive NK cells from donors with one or no copy of HLA-C2 (25). Both clinical and *in vitro* data suggest that NK cells that express activating KIR2DS1 are subjected to hyporesponsiveness via long-term contact of the activating KIR with its cognate ligand when two copies of the ligand are present. Whether or not NK cell hyporesponsiveness affects patient response in the context of ADCC, where mAb is administered to trigger NK cell activation through FcγRs, is still under investigation. As the interactions of activating KIRs and their ligands have not been studied as extensively as the interactions of the inhibitory KIRs and their ligands, much more characterization is required. Inhibitory KIR/KIR-ligand genotypes and activating KIR/KIR-ligand interactions may also serve as potential predictors of clinical outcome in the future.

### NK cell Fc receptor genetic variations

FcγRs function as receptors for the Fc portion of IgG immunoglobulins, and in doing so serve as a link of the innate immune system to the humoral system. In humans, there are three classes of FcγR, including variations of FcγRI, FcγRII, and FcγRIII. These three receptor classes are characteristically expressed on various immune cells. NK cells express both FcγRIIC and FcγRIIIA, which have low to intermediate affinity for IgGs (depending on FcR polymorphisms and IgG subclasses). As noted above, individuals with intact immune systems all express FcγRIIIA on most of their NK cells, less than half the population expresses FcγRIIC on their NK cells. Genetic variability exists within both FcγRIIC and FcγRIIIA, and this genotypic variation can vary the expression and avidity of these FcγRs for IgG molecules (185, 186).

As was mentioned above, several groups have found associations between SNP genotype of FcγRIII3A-158-V/F and patient response; those with the higher affinity V/V genotype respond better to some antibody therapies than those with V/F or F/F (42–46). In fact, FcγRIIIA-158-V has improved binding affinity for IgG1 subclasses as compared to FcγRIIIA-158-F, which is the most common IgG subclass used in mAb cancer immunotherapeutics. Interestingly, FcγRIIIA-158-V and FcγRIIIA-158-F have increased binding strength for IgG3 over IgG1 (187). This suggests that antibody IgG subclass variations may improve their interaction with NK cells, via improved engagement of FcγRIIIA, and thus improve tumor destruction. However, other FcRs (such as Fcwever) are expressed on other immune cells, such as neutrophils and monocytes/macrophages, that also play an important role in tumor killing, and have improved binding to IgG1 isotypes over IgG3 (188).

Another FcγR that is expressed on NK cells, that also has improved binding to IgG3 over IgG1, is FcγRIIC. FcγRIIC is the result of the unequal crossover of FcγRIIA (an activating FcR expressed on myeloid immune cell lineages) and FcγRIIB (an inhibitory FcR expressed on B cells, monocytes, and macrophages) (185). FcγRIIC also has SNPs within its nucleotide sequence that governs its expression (or non-expression) on the cell surface. SNP sites within exon 3 of FcγRIIC result in either an open reading frame, hence protein expression on the cell surface (FcγRIIC-ORF), or a stop codon, thus non-expression of FcγRIIC on the cell surface (FcγRIIC-STOP). As such, FcγRIIC is expressed in ~20–40% of the population, and co-expression of FcγRIIC with FcγRIIIA may result in enhanced ADCC capabilities of the NK cells (186).

Some of the FcγRs have been shown to be subject to copy number variability (CNV), and this CNV can result in variable expression of these FcRs on the cell surface. Both FcγRIIC and FcγRIIIA can be CNV, and CNV in these receptors correlates with differences in protein expression levels (189, 190), as well as increased ADCC function through enhanced NK cell activation (186, 190).

Given that the isotypes of FcγRIIIA have been shown to have differential binding affinity to IgG subclasses, that FcγRIIC expression on NK cells is variable within the population, and that these FcγRs can be CNV, using genotypic measures to pre-select patients based on their FcγR genotype for therapeutics that require NK cell may be of critical importance for future clinical investigations. Detailed simultaneous testing of polymorphisms and CNV in all three of these genes (FcγRIIA, FcγRIIC, and FcγRIIIA) has yet to be evaluated for associations with clinical outcome in clinical trials of ADCC-inducing tumor-reactive mAbs. However, it is likely that these factors, which should influence *in vivo* ADCC function, will be found to play a role in the clinical activity of ADCC-inducing mAb therapies.

## Concluding Remarks

Monoclonal antibodies utilize different mechanisms to destroy cancer cells, one of which is ADCC. As these treatments have continued to evolve from original mouse antibodies to chimeric antibodies to humanized and fully human antibodies, therapeutic mAbs for cancer treatment are still being engineered to achieve improved anti-tumor efficacy. Besides optimizing the characteristics of the mAb itself, there are other ways to improve anti-tumor effect, one of which is to improve ADCC by combining immune stimulatory therapies with the mAb. Since NK cells are considered a major player in mAb-mediated ADCC against tumor cells, reagents that can enhance NK cell activation, such as IL2, may be combined with mAb to improve the ADCC effect. However, due to IL2-associated toxicity in patients (191), novel IC therapeutics are being generated in an attempt to reduce toxicity and to allow for an increased MTD. Another cytokine that helps NK cell activation is IL15, and it is currently being tested clinically in various types of cancer. Besides cytokines or IC, newly discovered mechanisms that could potentially improve ADCC include MMP inhibitors to prevent FcR shedding, and ionizing radiation to make tumors more immunogenic and vulnerable to immune-mediated destruction. These approaches combined with tumor-specific mAb are still being explored in pre-clinical models to determine efficacy and optimize dosing regimens. Finally, genotypic profiles of NK cells also may contribute to our understanding of the magnitude of ADCC responses of NK cells in any given patient. Specifically, KIRs and FcR genotypes may help to predict clinical outcome to ADCC-inducing mAb therapy, allowing for more personalized treatment. More detailed analyses of associations of clinical outcome with NK cell genotype profiles are needed in order to determine the predictive value of this form of genotyping for distinct types of cancer and for different immunotherapies that involve NK cells.

## Conflict of Interest Statement

The authors declare that the research was conducted in the absence of any commercial or financial relationships that could be construed as a potential conflict of interest.
